# In Vitro Degradation of Electrospun Poly(Lactic-Co-Glycolic Acid) (PLGA) for Oral Mucosa Regeneration

**DOI:** 10.3390/polym12081853

**Published:** 2020-08-18

**Authors:** Ana Chor, Raquel Pires Gonçalves, Andrea Machado Costa, Marcos Farina, Arnaud Ponche, Lys Sirelli, Gautier Schrodj, Simon Gree, Leonardo Rodrigues de Andrade, Karine Anselme, Marcos Lopes Dias

**Affiliations:** 1 Biomineralization Laboratory, Institute of Biomedical Sciences, Federal University of Rio de Janeiro, Rio de Janeiro 21941-902, Brazil; anamedoral@gmail.com (A.C.); andreamachcosta@gmail.com (A.M.C.); marcos.farina.souza@gmail.com (M.F.); andrade@histo.ufrj.br (L.R.d.A.); 2 Institute of Macromolecules Professor Eloisa Mano, Federal University of Rio de Janeiro, Rio de Janeiro 21941-598, Brazil; raquelgoncalves.rp@gmail.com (R.P.G.); lys@ima.ufrj.br (L.S.); 3 The Mulhouse Materials Science Institute (IS2M), CNRS, University of Haute-Alsace, CNRS, UMR 7361, F-68100 Mulhouse, France; arnaud.ponche@uha.fr (A.P.); gautier.schrodj@uha.fr (G.S.); simon.gree@uha.fr (S.G.); karine.anselme@uha.fr (K.A.); 4 University of Strasbourg, F-67081 Strasbourg, France

**Keywords:** biomaterials, electrospinning, in vitro degradation, oral mucosa, PLGA, saliva, simulated body fluid

## Abstract

Poly(lactic-co-glycolic acid) (PLGA) has been used in the field of tissue engineering as a scaffold due to its good biocompatibility, biodegradability and mechanical strength. With the aim to explore the degradability of PLGA electrospun nonwoven structures for oral mucosa tissue engineering applications, non-irradiated and gamma irradiated nonwovens were immersed in three different solutions, in which simulated body fluid (SBF) and artificial saliva are important for future oral mucosa tissue engineering. The nonwovens were immersed for 7, 15 and 30 days in SBF, culture media (DMEM) and artificial saliva at 37 °C. Before immersion in the solutions, the dosage of 15 kGy was applied for sterilization in one assay and compared with non-irradiated samples at the same timepoints. Samples were characterized using different techniques such as scanning electron microscopy (SEM), differential scanning calorimetric (DSC) and gel permeation chromatography (GPC) to evaluate the nonwoven degradation and Fourier-transform infrared spectroscopy (FTIR) to evaluate the chain scissions. Our results showed that PLGA nonwovens were constituted by semicrystalline fibers with moderate degradation properties up to thirty days. The non-irradiated samples exhibited slower kinetics of degradation than irradiated nonwovens. For immersion times longer than 7 days in the three different solutions, the mean diameter of irradiated fibers stayed in the same range, but significantly different from the control sample. On non-irradiated samples, the degradation kinetics was slower and the plateau in the diameter value was only attained after 30 days of immersion in the fluids. Plasticization (fluid absorption into the fiber structure) occurred in the bulk material, as confirmed by a decrease in Tg observed by DSC analyses of non-irradiated and irradiated nonwovens, in comparison with the respective controls. In addition, artificial saliva showed a higher capacity of influencing PLGA crystallization than SBF and DMEM. FTIR analyses showed typical PLGA chemical functional groups changes. These results will be important for future application of those PLGA electrospun nonwovens for oral mucosa regeneration.

## 1. Introduction

Tissue engineering is an interdisciplinary field, which combines specific cell types and engineered scaffolds for tissue regeneration. The term “tissue engineering” was crafted in the early 1980s [1] and since then, various scaffolds have been produced to simulate the extracellular environment for different tissues and organs [2,3].

In the 1990s, temporomandibular joint regeneration was reported and since then, tissue engineering approaches have been used to the regeneration of craniofacial regions and applications in dentistry for soft tissues regeneration, such as the periodontal ligament, pulp, oral mucosa and salivary gland, as well as hard tissues, such as enamel, dentin, cementum and alveolar bone. In all reports, polymers have been used to guide these hard and soft tissue regenerations in preclinical and clinical studies [2,4].

For clinical applications, new polymer scaffolds need to be previously tested in vitro and in animal models to achieve the best parameters in terms of biocompatibility, specific mechanical properties, optimized porosity for nutrients flow and biodegradability rate [4,5]. Among polymers, aliphatic polyesters such as poly(lactic acid) (PLA), poly(glycolic acid) (PGA) and their copolymer poly(lactic-co-glycolic acid) (PLGA) have been chosen because of their excellent biocompatibility and controllable biodegradability [6,7]. In the body, these polymers are degraded through the cleavage of ester bonds, and the byproducts metabolized by cells or excreted in the urine [8]. In the case of copolymers like PLGA, polymers with different monomer’s composition can provide materials with different stiffness and degradation rates in vitro and in vivo. All these polymers have been used for tissue engineering in dentistry, in addition to natural polymers such as chitosan, collagen and cellulose [9].

Among the methods used to produce oral mucosa replacements, electrospinning is attractive due to the possibility of producing 2D or 3D biomaterials with open porous structures, with similarities to the structure of the collagenous dermis. A good example was reported by Blackwood et al. [10] who developed biodegradable electrospun scaffolds for dermal and oral mucosa replacement. Although tissue engineering of oral mucosa is less developed than skin tissue engineering, Kumbar et al. [11] highlighted the progress of tissue engineering of dental hard and soft tissues such as enamel, dentin, alveolar bone, periodontium, oral mucosa and salivary glands.

The electrospinning method produces aligned or randomly organized micro- or nano-fibers into thin nonwoven (2D) or thicker scaffolds (3D) [12]. Electrospun scaffolds have been tested for different biomedical applications [6,12], such as in bone, skin, tendon and cornea reconstruction [7,11,12,13,14,15,16].

Blackwood and coworkers [10] have examined electrospun polymer scaffolds of PLA and PLGA with a different composition of the biodegradable replacement dermal substitute for tissue engineering of skin and oral mucosa. Samples were evaluated for degradation in vitro and in vivo. They found that electrospun scaffolds permitted good cellular penetration, with no adverse inflammatory response outside the scaffold margin. The scaffolds fabricated from PLGA 85:15 revealed a 50% loss of mass after 4 months, and breakdown rate similar for in vitro and in vivo experiments. No in vitro experiment was carried out using saliva.

It is interesting to highlight that in oncology area patients with head and neck cancers under chemo and radiotherapy often develop oral mucositis as a side effect [16]. Oral mucositis causes multiple ulcers in the oral mucosa, which in many times inhibit the patients feeding. The use of biodegradable and biocompatible membranes might improve the ulcers regeneration in shorter times compared to the classical approach, thus avoiding prolonged interruption of the cancer treatment.

For application of polymer scaffolds in dentistry, it is important to know the material behavior in saliva. Nevertheless, few studies using simulated saliva have been reported. The in vitro degradation and swelling behavior of rubbery thermoplastic starch in simulated body and saliva fluid was previously reported and the effects of degradation products on cells analyzed by Shi and coworkers [17]. Another publication on the subject was reported by Liao et al. [18], who investigated the degradation in artificial saliva of layered nano-carbonated hydroxyapatite/collagen/PLGA composite casting membranes in vitro. Recently, degradation of menthol-loaded PLGA micro and nanospheres in artificial saliva was also reported [19]. So, as far as we are concerned, no report of degradation in artificial saliva of PLGA nonwoven structures for dentistry application was reported. Thus, in this work we present the results of the comparative study of degradation of electrospun PLGA nonwoven in simulated saliva. PLGA 85:15 nonwovens were prepared by electrospinning, sterilized by gamma radiation and their in vitro degradation tested using simulated body fluid (SBF), culture media and artificial saliva up to 30 days. SBF was chosen as a reference solution to study biomaterials degradation over time [20,21]. Since PLGA scaffolds can be loaded with cells in a tissue engineering approach to improve tissue regeneration, testing its degradation rate in culture media is an important step. Finally, since our future goal is to treat oral mucosa lesions with PLGA nonwoven, understanding the influence of saliva on membranes degradation is also crucial.

In terms of analytical techniques to follow in vitro degradation, we applied scanning electron microscopy (SEM) to evaluate the fibers morphology, differential scanning calorimetry (DSC) to evaluate the nonwoven thermal properties, gel permeation chromatography (GPC) to determine the polymer molecular weight changes and Fourier transform infrared spectroscopy (FTIR) to identify possible changes in PLGA typical chemical functional groups.

## 2. Experimental

### 2.1. Materials

Poly(L-lactic-co-glycolic acid) (Purasorb PLGA 85:31) 85:15 molar ratio with inherent viscosity of 3.1 dL/g was purchased from Corbion-Purac (Gorinchem, The Netherlands). Chloroform (CHCl_3_) (Sigma-Aldrich, Saint louis, MO, USA) and dimethylformamide (DMF; Sigma-Aldrich) were used as solvents for electrospinning. The SBF solution was prepared according to Kokubo and Takadama [21]. Cell culture media (Dulbecco’s Modified Eagle’s Medium - DMEM low glucose D6046) was purchased from Sigma-Aldrich and artificial saliva produced by University Pharmacy from Federal University of Rio de Janeiro (Brazil; Table S1 Supplementary Materials).

### 2.2. Electrospinning of PLGA Nonwoven

To perform the electrospinning process, polymer solutions were prepared by dissolving 5% of PLGA (w/v) in binary-solvent systems of CHCl_3_ and DMF (80/20 *v*/*v*). All solutions were magnetically stirred at room temperature until complete powder dissolution confirmed by a transparent fluid. A syringe with a 20-gauge needle containing 5 mL of 5% PLGA was attached to a pump (KDS 100 Infusion Syringe Pump) and pumped at a flow rate of 0.6 mL/h. Electrospinning was carried out at 15 kV by using a Glassman High Voltage, PS/FC 60p02.0-11 source and a working distance of 15 cm from the needle tip to the collector was used. A total spinning time of 6 h was employed to produce nonwoven structures with 10 × 10 cm size. Preliminary condition tests to find the adequate voltage, distance and polymer concentration used in this work was previously performed to optimize fiber morphology.

During the electrospinning process, a high electric field is applied on a polymer solution, which causes a cone-shaped deformation of the polymer solution drop, known as Taylor’s cone, in the direction of the counter electrode. Once the electric field overcomes the surface tension of the solution cone, a charged jet is ejected towards the collector. Along the way, the diameter of the jet decreases as the result of solution stretching at the same time the solvent is evaporated. As a consequence, random fibers are formed and deposited in a plate-like collector [6,11].

Although the spinning solutions were prepared by the using solvent mixture containing chloroform, which is known to have a certain degree of toxicity, electrospun fibers contain practically no residual solvent after being formed by electrospinning since it is completely evaporated during jet flying. In addition, in this work eventual residual solvent was removed by washing the nonwoven structures with distilled water.

### 2.3. Membranes Sterilization

The nonwovens were sterilized by gamma rays from a cobalt 60 source (MDS Nordion GC 220 E model, from Laval, Que, Canada) with a total dosage of 15 kGy [22,23]. Since 10–30 kGy is reported to promote the desired sterilization effect, this dosage was chosen in the present work aiming the preservation of the structural integrity of the electrospun nonwoven.

### 2.4. In Vitro Degradation Tests

Gamma irradiated and non-irradiated PLGA nonwoven of 3 cm × 5 cm were transferred to 3 cm-plastic sterile petri dishes and immersed in 10 mL of SBF, DMEM and artificial saliva (Table S1). The dishes were placed in a cell incubator at 37 °C, 5% CO_2_ and 95% humidity for 7, 15 and 30 days. The tested solutions were changed every 7 days. After each time point, the nonwovens were removed, washed 3× for 10 min in distilled water; 3 times in absolute ethanol for 10 min, dried at room temperature and stored at 4 °C for future analysis. This study focused on the degradation of PLGA 85:15 for 4 weeks to observe maintenance of the structure integrity after 2 weeks, which is important to induce cell proliferation in the injured site [10].

### 2.5. Nonwoven Characterization

For SEM analysis, dried nonwovens were sputter-coated with a thin gold layer (4 nm) (BAL-TEC SCD 050, Capovani Brothers, Scotia, NY, USA), and their surface morphology examined on a QUANTA 250 (Fei, Hillsboro, OR, USA), operated at 15 kV. The samples were gold metallized for observation (2048 pixels × 2048 pixels). Five SEM images were randomly acquired for each condition at 1000× magnification and at least the diameter of 10 fibers per image was measured using Fiji-ImageJ program (NIH). Microsoft Excel program was used to determine the mean, standard deviation and size distribution.

The thermal properties of the PLGA nonwovens were investigated by DSC (METTLER-TOLEDO DSC1, Mettler-Toledo SAS, Viroflay, France), which determines the heat energy consumed or radiated by a specific sample [24]. We used two heating cycles from 0 to 300 °C at 10 °C/min, intercalated with cooling from 300 to 0 °C at 10 °C/min, with a 5 min isothermal step between each cycle (heating–cooling–heating). From these heating and cooling cycles, data on glass transition temperature (*T*_g_), melting temperature (*T*_m_), crystallization temperature (*T*_c_) and degree of crystallinity were obtained. The degree of crystallinity (*X*_c_) was calculated as the ratio between enthalpies, following the equation: *X*_c_ = (Δ*H*_m_ − Δ*H*_cc_) 100/Δ*H*_m_°, where (Δ*H*_m_) is the fiber melting enthalpy, Δ*H*_cc_ is cold crystallization enthalpy and Δ*H*_m_° is the melting enthalpy of a theoretically 100% crystalline PLA 100% crystalline PLA (Δ*H*_m_° = 106 J/g) [25].

The variation in molecular weight between the control (recent produced) and the degraded membranes, sterilized or not by gamma radiated, were obtained by GPC using a Shimadzu LC 20 instrument (Riverwood Drive, Columbia, MD, USA) equipped with a Shim-pack GPC-804 column (Shimadzu, Riverwood Drive, Columbia, MD, USA) and a RID-20A differential index detector (Shimadzu, Riverwood Drive, Columbia, MD, USA). The analyses were performed using CHCl_3_ as mobile phase with a flow rate of 1 mL/min at 25 °C. Polystyrene standards were used for calibration. Molecular weight average number (*M*_n_), weight-average molecular weight (*M*_w_) and the index of dispersity (*Ð*) were obtained [26].

Changes in the chemical structure of polymer fibers were characterized by FTIR using the attenuated total reflection mode (FTIR-ATR) in a DIGILAB FT5 3000 spectrometer (Varian, Atlanta, GA, USA) and IFS66/S (Brucker, Marne-la-Vallée, France), equipped with an ATR Zn Se crystal. Spectra were obtained in absorbance mode using Win-IR Pro 3.3 software, with wavenumbers range between 500 and 4000 cm^−1^ and with 4 cm^−1^ resolution. The spectra of irradiated and non-irradiated PLGA membranes were normalized considering the Y absorption values from 868 cm^−1^ band attributed to the C–C (O) O stretching mode [27]. The Origin 7.0 program was used for normalization and the graphs.

### 2.6. Statistical Analysis

The fibers diameter results were analyzed with Origin Pro software (OriginLab, Northampton, MA, USA, www.originlab.com) using a one-way analysis of variance (ANOVA). Diameter means after immersion in buffers were further compared according to the Tukey’s post hoc test (α = 0.05). The Tukey test compared fiber diameters between two time points and conditions.

## 3. Results

### 3.1. Morphology of Electrospun PGLA Fibers

Since all implantable grafts should be sterilized after use, the process should not interfere or modify the polymer chemical properties. Gamma radiation is a common method for sterilization with high penetration, being a worldwide acceptable technique for implantable devices [2,22,23].

**Figure 1:**
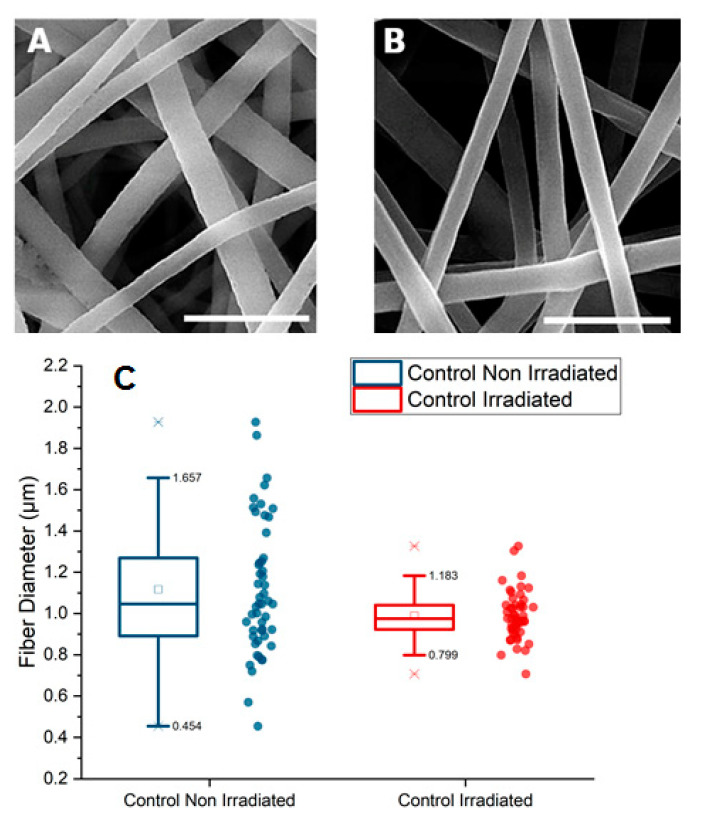
SEM images of control electrospun PLGA nonwoven before in vitro degradation: (**A**) non-irradiated, (**B**) irradiated (Scale bar: 5 µm) and (**C**) distribution of fibers diameter (mean value of the diameters is represented by a square and median value by a horizontal line in the box).

Firstly, we investigated the influence of γ-rays on the morphology of PLGA fibers by SEM. Figure 1A,B present non-irradiated and γ-irradiated PGLA nonwoven with random organized fibers with round edges and smooth surfaces. Those fibers before immersion in different fluids were considered as a control. We measured the fibers diameters for both conditions and found similar mean values for non-irradiated (1.1 ± 0.3 µm) and irradiated (1.0 ± 0.1 µm; Figure 1C). The most significant result here is the dispersion of the diameters values before irradiation and the strong narrowing of the distribution after irradiation. The difference between the two populations of fibers was statistically different (*p* = 0.01058). Molecular weight loss after gamma irradiation due to chain scission probably promoted narrowing of the fibers diameter.

**Figure 2:**
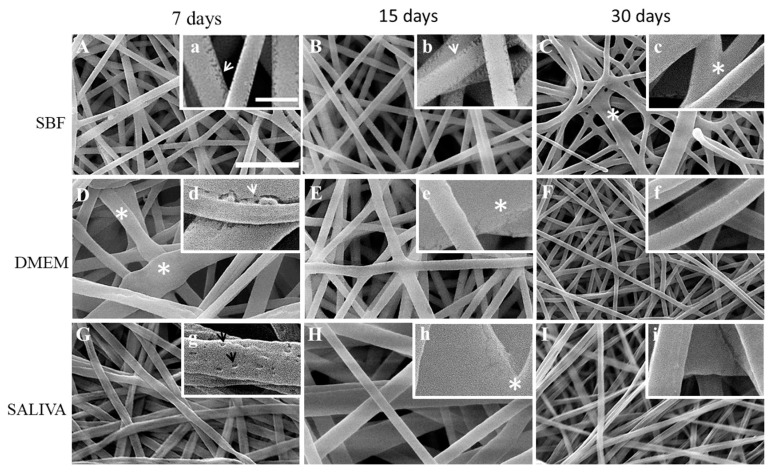
Representative SEM micrographs of non-irradiated electrospun PLGA after immersion in simulated body fluid (SBF), DMEM and saliva for 7, 15 and 30 days. Scale bars: (**A**–**I**) = 5 µm; (**a**–**i**) = 1 µm. Fibers morphology after 7 and 15 days of immersion in SBF showed borders fissures (**a,b, arrows**); after 30 days of immersion in SBF, border fissures were less evident and fibers showed connections to each other (**C,c, asterisks**). After 7 days of immersion in DMEM, fibers swelled (**D, asterisks**), and showed an area with an elongated fissure (**d, arrow**); after 15 days of immersion in DMEM, fibers connected to each other due to hydration (**e, asterisks**); after 30 days of immersion in DMEM, fibers narrowed and connected to each other, and showed scarce border fissures (**F**). After 7 days of immersion in saliva, fibers showed shrinkage (**G**), and surface pores (**g, black arrows**); after 15 days of immersion, fibers showed to have swollen (**H**) and joined each other, with scarce border fissures (**h, asterisks**); after 30 days of immersion, fibers narrowed and showed no evidence of pores (**I,i**).

**Figure 3:**
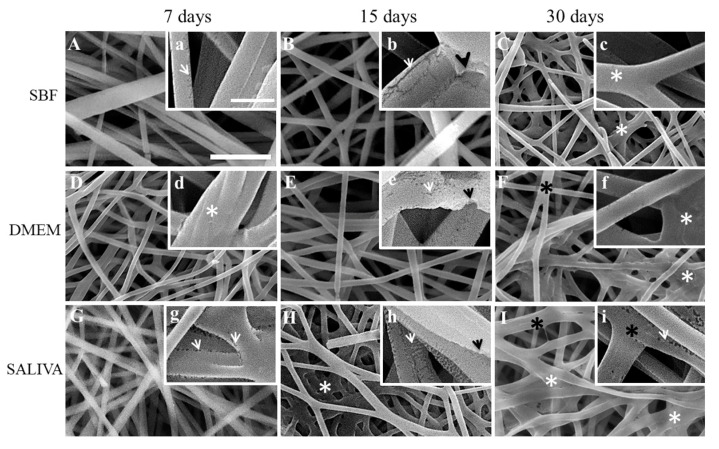
Representative SEM micrographs of irradiated electrospun PLGA after immersion in SBF, DMEM and saliva for 7, 15 and 30 days. Scale bars: (**A**–**I**) = 5 µm; (**a**–**i**) = 1 µm. Membrane immersed in SBF for 7 days showed fibers with border fissures. (**a, arrow**); Elongated fissures were seen in different regions of fibers after 15 days of immersion in SBF (**b, white arrow**) and also erosion areas (**b, black arrow**); after 30 days of immersion in SBF, fibers became in more contact to each other (**C, c, asterisk**). Morphology of fibers after immersion in DMEM for 7 days shows different directions of fibers and connections to each other (**d, asterisk**); After 15 days of immersion in DMEM, fibers showed surface fissures (**e, white arrow**) and erosion areas (**e, black arrow**). Morphology of fibers after 30 days of immersion in DMEM showed coalescence areas (**F,f, white asterisks**) and also connected fibers (**F, black asterisks**). Fibers showed border fissures after 7 days of immersion in saliva (**g, white arrow**); after 15 days, fibers showed increased border and surface fissures (**h, white arrow**) and erosion areas (**h, black arrow**); after 30 days of immersion in saliva, fibers showed scarce border fissures (**i, white arrow**), coalescence areas among fibers (**I, white asterisks**) and also fibers junctions (**I,i, black asterisks**).

Further, non-irradiated (control; Figure 2) and gamma irradiated (Figure 3) PLGA nonwoven treated by SBF, DMEM and artificial saliva for 7, 15 and 30 days were observed by SEM. Non-irradiated nonwoven immersed in SBF presented fibers with surface defects like microcracks, suggesting that immersion caused some degree of degradation in the fibers after 7 and 15 days comparing to the fibers before the treatment (Figure 2A,a,B,b). Nonwovens immersed in DMEM did not show marked evidence of fiber microcracks or fissures (Figure 2D,d) compared with SBF fibers. Nonwovens exposed to artificial saliva showed fibers with many concavities on the surface after 7 days Figure 2G,g that were not seen at 15 and 30 days (Figure 2H,h,I,i). From 15 to 30 days fibers coalescence and shrinkage occurred (Figure 2I).

Gamma irradiated nonwoven immersed in the three solutions are presented in Figure 3. After 15 days in SBF, it is possible to observe an enlargement of the fissures in irradiated fibers and a connection to each other (Figure 3B) In addition, connections among fibers were observed, and the initiation of coalescence (Figure 3C,I).

**Figure 4:**
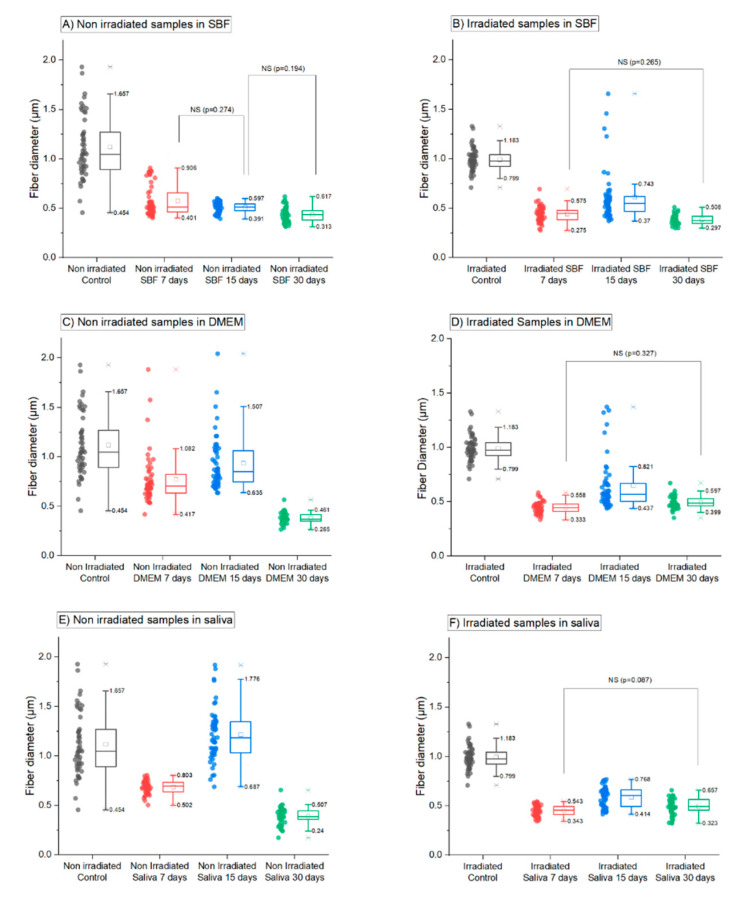
Distribution of fibers diameter for non-irradiated (left) and gamma irradiated PLGA fibers (right) after in vitro degradation during 30 days of immersion in SBF (**A**,**B**), DMEM (**C**,**D**) and saliva (**E**,**F**) (the mean value of the diameters is represented by a square and median value by a horizontal line in the box). NS indicates that the difference of the means is not significant at the 0.05 level (probability specified in bracket and calculated via a Tukey test). If nothing is specified, the difference of the means is significant at the 0.05 level for a pair of samples.

The quantitative analysis of the distribution of fiber diameters after ageing in three different solutions (saliva, DMEM or SBF solutions) after 7, 15 and 30 days is presented in Figure 4. It confirmed that irradiated and non-irradiated samples behaved differently after immersion in the three solutions. For non-irradiated samples, effect on the fiber diameter was dependent on the time and type of fluid whereas irradiated samples exhibited an identical decrease in the fiber diameter already after 7 days of immersion in the three solutions. All samples (irradiated or non-irradiated) immersed in the three solutions exhibit significant differences of the means at a *p* = 0.05 level compared to the control, except for one population: non irradiated samples immersed in saliva for 15 days. The hydrolysis process has an immediate effect on the diameter measured in all the samples. Nevertheless, the decrease in the diameter is more pronounced after a shorter immersion time on irradiated samples. The diameter means attained a plateau around 0.45 µm after 7 days on irradiated samples (Figure 4B,D,E) whereas it takes 30 days to attain this value on non-irradiated samples immersed in DMEM and saliva (0.381 ± 0.007 µm after 30 days in DMEM solution and 0.39 ± 0.01 µm after 30 days in saliva).

In more details, for non-irradiated samples the process of reduction of the diameter was progressive and solution dependent. In SBF, a strong decrease (about 52%) in the mean diameter value and a large distribution of diameters were observed after immersion for 7 days while a smaller decrease (about 10%) was observed after 15 days (Figure 4A). For the DMEM solution, the significant decrease in the mean diameter value was only observed after 30 days (about 60%). Only a limited decrease could be observed after 7 and 15 days (about 20% compared to control samples), but it was statistically significant compared to the control (Figure 4B). In saliva, a strong decrease in the mean diameter with a lower diameter distribution was only observed after immersion for 30 days (about 30%).

For irradiated samples, immersion in SBF, DMEM and saliva solution had a significant decrease (about 55%) in the mean diameter at the early stage (7 days). For example, the mean value decreased from 1.0 ± 0.1 µm for the irradiated control samples to 0.44 ± 0.05 µm for immersion in the DMEM solution for 7 days. For the three solutions and immersion time longer than 7 days, the mean diameter value had a small variation (about 30%; see Figure 4B,D,F) and was significantly different from the control sample (*p* < 0.001 for the two sample post hoc Tukey test). The comparison of diameters between non-irradiated and irradiated samples immersed in the three different fluids indicated that the kinetics of degradation was generally slower on non-irradiated samples than on irradiated ones.

### 3.2. Molecular Weight Analyses

Table 1 and Table 2 present the average molecular weights of PLGA non-irradiated and irradiated samples, after immersion in the three different solutions. It is important to mention that some nonwoven did not dissolve completely in chloroform for the GPC analysis, and in these cases, only the soluble fraction was analyzed.

As shown in Table 1, the control non-irradiated PLGA presented higher molecular weights (*M*_n_ = 253,900 and *M*_w_ = 460,400) while the irradiated membranes (Table 2) showed a significant decrease in the molecular weights (*M*_n_ = 75,000 and *M*_w_ =156,300), revealing that sterilization by gamma irradiation caused a strong PLGA degradation (66% of *M*_w_ lost). High molecular weight polymers, as the non-irradiated PLGA used in the present study, have long chains, being more susceptible to the drastic decrease of average molecular weight when exposed to hydrolytic fluids [28]. In this regard, a drastic decrease in the molecular weight of the non-irradiated nonwoven immersed in saliva at 30 days was observed (Table 1) when compared to the irradiated nonwoven immersed in saliva at the same time point, both relative to each control (first lines Table 1 and Table 2). The slight increase in *M*_n_ and *M*_w_ observed for two samples after immersion at 15 days in SBS and DMEM is under the expected uncertainty for the GPC technique and, since there is no reason for such an increase, it was considered an experimental error. Considering mass loss during degradation in solutions, Perron et al. showed that the percentage of water absorption after immersion of PLGA (85:15) in phosphate buffer saline (PBS) and distilled water (dH_2_O) after 84 day, were solution dependent: (i) after immersion in PBS solution, scaffolds water absorption increased from 0 to 100% after 7 days and remained constant for the other periods of degradation and (ii) the water uptake of the scaffold exposed to dH_2_O varied from 0 to 150% after 7 days of degradation and increased up to 200% towards the end of the degradation study [28]. Authors observed that mass loss was within 4% throughout the full degradation period, and as mass loss water uptake decreases [29].

### 3.3. Thermal Behavior of Irradiated and Non-Irradiated PLGA Nonwovens

Electrospun PLGA control nonwoven, gamma irradiated or not, and the nonwoven subjected to the degradation process in solution were analyzed comparatively by DSC. Table 3 and Table 4 present the values of the main thermal transitions and degree of crystallinity for the analyzed nonwoven after 7, 15 and 30 days of immersion in SBF, DMEM and artificial saliva.

**Figure 5:**
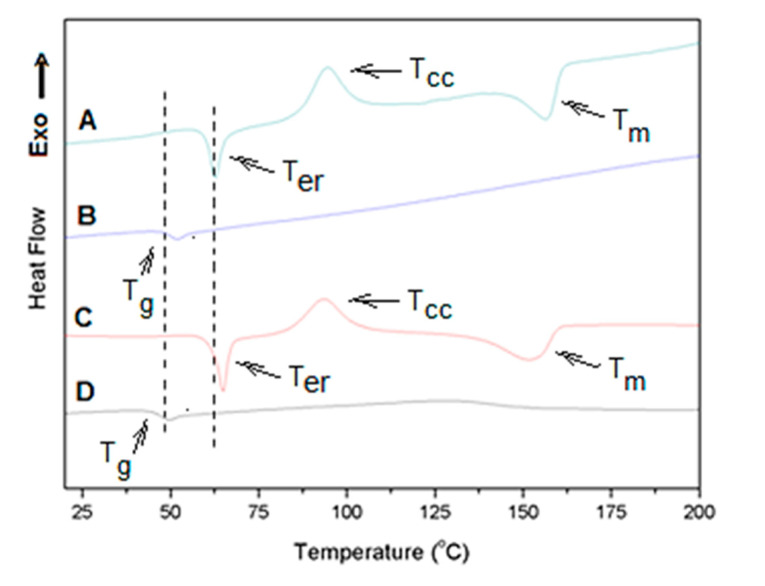
Differential scanning calorimetry (DSC) traces of non-irradiated and irradiated control PLGA nonwovens. (**A**,**B**) First heating run and second heating run, respectively, of non-irradiated membranes and (**C**,**D**) first and second heating run, respectively, of irradiated membranes.

The DSC curves of all non-irradiated and irradiated nonwovens showed exothermic and endothermic events in the first heating run before and after immersion in the fluids. Figure 5 shows representative DSC profiles for these nonwovens. These events are compatible with crystallization and crystalline melting phenomena, confirming that the PLGA used in this work contains a high content of L-lactic acid isomers sequences capable of crystallizing [30].

The first DSC heating run shows the sample thermal history and provides information on the crystalline state of the nonwoven after the electrospinning and irradiation processes. In addition, it also shows the effect of the fluids on the crystallinity along the immersion time.

The curve of Figure 5 shows a very anomalous behavior with two endothermic and one exothermic event. The first endothermic event appears as a peak at 64.1 °C (6.36 J/g; Figure 5A). This event is close to the temperature expected for the glass transition of high molecular weight lactic acid homopolymers, and it could be attributed to the known enthalpic relaxation phenomenon, which usually appears in the region of glass transition. Enthalpic relaxation has been reported for some amorphous electrospun fibers of semicrystalline polymers, including lactic acid polymers. It has been associated with energy absorption due to stress relaxation of oriented chains, which are produced by the high stretching of chains during electrospinning process [31]. Our data showed this first endothermic event only in the first DSC heating run and as a peak at about 20 °C above *T*_g_ determined from a second heating of the material (*T*_g_ around 46 °C; Figure 5B,D) which appeared as the classical glass transition curve profile. So, although surprisingly this peak appeared almost 20 °C above the *T*_g_, this endothermic event seems to be associated to the enthalpic relaxation process of the amorphous phase produced due to the high level of chain stretching induced during the electrospinning process.

An exothermic event was also seen in the first heating of the membrane after the enthalpic relaxation temperature peak (*T*_er_; Figure 5A). This event can be attributed to fiber crystallization during the heating cycle (cold crystallization) and it is evidence that fibers were predominately amorphous before the heating since they could be thermally crystallized. The non-irradiated PGLA fibers presented a cold crystallization temperature *T*_cc_ at 93.5 °C (Δ*H*_cc_ = 12.5 J/g; Table 3, footnotes). After the cold crystallization, the formed crystals melt, presenting a melting temperature *T*_m_ at 152.0 °C (Δ*H*_m_ = 16.4 J/g and *X*_c_ = 3.7%; Figure 5A and Table 3).

Figure 5C shows the first heating run DSC trace for the irradiated PLGA nonwoven. The curve had a similar profile as in Figure 5A (non-irradiated). It means that the fibers also presented a first endothermic process *T*_er_ (Δ*H_er_* = 7.06 J/g; see also Table 4), followed by a cold crystallization and melting of thermally formed crystals during heating. Irradiation provoked a slight increase in the transition temperatures and decrease in the degree of crystallinity (footnotes, Table 3 and Table 4), indicating that irradiation caused changes in the thermal behavior of the fibers that form the membranes.

Table 3 shows the transition temperatures, enthalpies and degree of crystallinity (*X*_c_) obtained from DSC curves of non-irradiated nonwovens after immersion in SBF, DMEM and artificial saliva. All DSC curves also presented the set of endothermic and exothermic events observed for the non-irradiated and irradiated nonwoven controls. In some cases, the curves showed a higher profile complexity, as could be seen for the non-irradiated PLGA nonwoven immersed in SBF for 7 days. In this nonwoven an endothermic event before glass transition and a double crystal melting *T*_m_ was observed. In general terms, all the non-irradiated nonwovens presented the endothermic event of enthalpic relaxation with peak temperature (*T*_er_) between 62 and 70 °C and Δ*H_er_* enthalpies ranging from 5 to 18 J/g. This means that by immersion in the different fluids, the level of stress relaxation of oriented chains was modified by the fluid penetration. It was not possible to observe any tendency or relation between *T*_g_ and *T*_cc_ with the interaction time and fluid type. *T*_g_ occurred between 43 and 49 °C, and *T*_cc_ between 92 and 96 °C. The non-irradiated nonwovens immersed for 7 days in SBF and DMEM showed a double crystal melting *T*_m_, and formation of high melting temperature crystals. For example, when immersed for 7 days in SBF, another peak of *T*_m_ at 182.4 °C was observed. This means that immersion in SBF and DMEM induced crystallization and formation of these high *T*_m_ crystals.

Transition temperatures, enthalpies and degree of crystallinity obtained from DSC curves of irradiated nonwovens after immersion in SBF, DMEM and artificial saliva, are shown in Table 4. No dramatic difference in the thermal behavior could be identified between irradiated and non-irradiated nonwovens, except for the enthalpies relaxation events for the nonwovens immersed in artificial saliva. This event was much higher than those of the controls and those found by immersion in the other fluids (SBF and DMEM). In this case, a clear increase in Δ*H_er_* was observed as the contact time increased, reaching an enthalpy of 22.9 J/g in the irradiated nonwoven immersed for 30 days, which was higher than the enthalpy of crystals formed by the electrospinning (control, Δ*H*_m_ = 0.8 J/g, *X*_c_ = 0.8%). For these irradiated nonwovens, the degree of crystallinity showed an opposite behavior relating to the non-irradiated ones. The crystallinity increased with the immersion time, indicating the effect of the fluids in inducing post-crystallization.

### 3.4. FTIR Spectroscopic Characterization of PLGA Nonwovens Before and After Gamma Irradiation

FTIR spectroscopy was used to identify changes of typical chemical functional groups of the PLGA polymer. PLGA presents typical vibrational modes in the infrared region like aliphatic C-H stretching modes (3000–2850 cm^−1^), carbonyl C=O stretching modes (1850–1650 cm^−1^), CH_3_/CH_2_ symmetric angular deformation (1500–1250 cm^−1^) and ester C–O asymmetric stretching modes (1300–1000 cm^−1^) [32].

**Figure 6:**
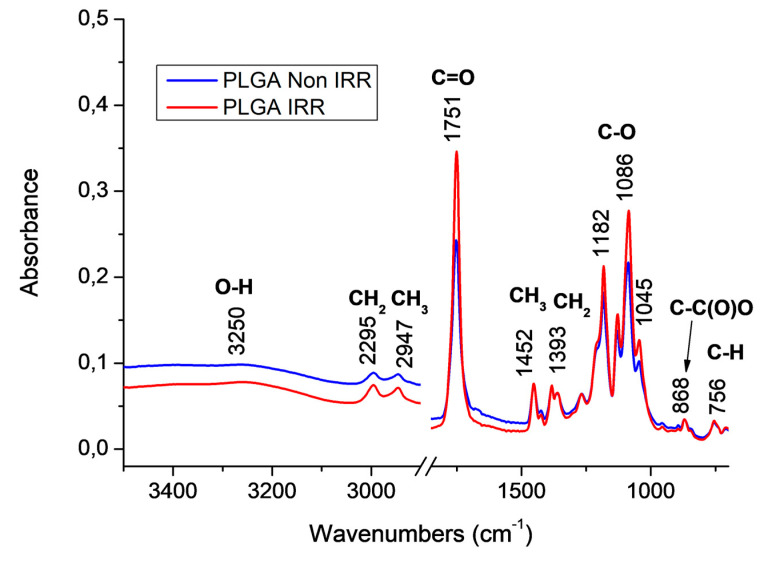
Normalized FTIR using the attenuated total reflection mode (FTIR-ATR) spectra of PLGA nonwovens before and after gamma irradiation.

Figure 6 shows the normalized FTIR-ATR spectra of electrospun poly (L-lactic-co-glycolic acid) (80:15) nonwovens before (in blue) and after gamma irradiation (in red). The spectra of PLGA irradiated (PLGA IRR) and non-irradiated (PLGA Non-IRR) nonwovens presented the PLGA characteristics bands with similar intensities. It was also noted a weak broad band related to stretching vibrational modes of OH groups (3250–3300 cm^−1^). This band could be related to water absorption on the nonwoven surface. Although the moisture content of PLGA is low at room temperature, a high relative humidity could reach 0.6% of water on PLGA films [33].

**Figure 7:**
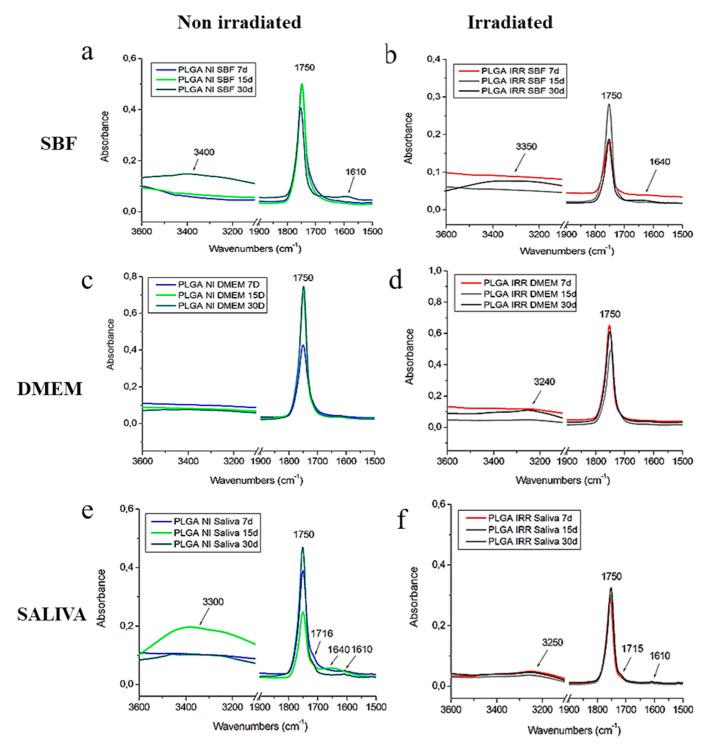
Normalized FTIR-ATR spectra of PLGA non irradiated and irradiated nonwovens after 7, 15 and 30 days immersed in SBF, DMEM and artificial saliva at 37 °C: (**a**,**c**,**e**) gamma non irradiated PLGA. (**b**,**d**,**f**) Gamma irradiated PLGA.

Normalized FTIR-ATR spectra of irradiated and non-irradiated PLGA nonwovens after immersion in the three solutions (SBF, DMEM and artificial saliva) at 37 °C during 7, 15 and 30 days are shown in Figure 7. For samples immersed in SBF solutions, an increase on OH stretching modes was observed after 30 days suggesting more water absorption on both samples after 30 days. It is also important to note the additional band at 1610 cm^−1^ for PLGA non-irradiated nonwoven (Figure 7a). Lee et al. studied the degradation process of PLGA (50:50) nanofibers exposed to e-beam radiation and immersed in PBS solution [34]. According to these authors, an asymmetric stretching mode of O=C–ONa groups at 1600 cm^−1^ is attributed to a mixture of acidic oligomers from the PLGA degradation and sodium ions from PBS. This fact could explain the band at 1610 cm^−1^ found in this case [34].

For PLGA irradiated nonwoven immersed on SBF (Figure 7a) an additional band found at 1640 cm^−1^ could be attributed to O-H groups from water [35]. PLGA nonwovens immersed in DMEM solution (Figure 7c,d) presented similar profiles when compared with the control PLGA nonwovens, i.e., before immersion (Figure 6). These results suggested a low degradation in this case, since additional bands were not observed. On the other hand, the immersion in the saliva solution causes more differences on the FTIR spectra of PLGA nonwovens with more similarity to SBF results, which showed an increase of OH stretching modes and additional bands (Figure 7a (1610 cm^−1^) and Figure 7b (1640 cm^−1^)). Additional bands at 1715, 1640 and 1610 cm^−1^ was observed, in the non-irradiated membrane (Figure 7e) and in the irradiated nonwoven immersed in saliva (1715; 1610 cm^−1^), suggesting a higher degradation in this case. The band found at 1715 cm^−1^ seems to be related to the hydrogen bond in water, coordinated with the polymer carbonyl group (C=O) from a shift on C=O vibration modes [36]. These results indicated a polymer chemical change probably due to the hydrolysis degradation of PLGA nonwovens immersed in the saliva solution.

## 4. Discussion

The dispersions in fiber diameters presented in our work demonstrate similarities with the works of Blackwood et al. [10] and Gilcastell et al. [37] in which variations in fiber diameters were shown before and after different fluid treatments. It is also important to mention that changes in morphology and the diameter of fibers may be influenced by small changes during the electrospinning process like voltage. As an example, You et al. [38] described that the chloroform used as a solvent for electrospun PLGA caused a high average on fiber diameter (760 nm), with a broad distribution of the fibers, varying from 200 to 1800 nm.

We analyzed the in vitro degradation of electrospun degradable PLGA nonwoven in three different solutions as a preliminary step for its use in oral mucosite ulcer regeneration in vivo. We choose the PLGA 85:15 proportion because the polymer exhibits an intermediate degradation rate comparing with homopolymer based materials. Indeed, You et al. found that the degradation rate for the polyesters were: PGA > PLGA (50:50) > PLA for different experimental conditions used for the electrospinning process [38]. The biodegradation rate is influenced by a variety of parameters including crystallinity, molecular weight, copolymer composition, morphological structure, polymer *T*_g_ value and the type of fluid in contact with the polymers [38,39]. Considering the last mentioned parameter, we postulated that the three chosen fluids were pertinent to the use of PLGA scaffolds for oral mucosa.

Since all scaffolds must be sterilized for animal and human uses, we first focused on the influence of gamma irradiation on the physicochemical properties of electrospun PLGA microfibers. We observed the fibers morphology by SEM and measured their diameters under all conditions. The average diameters for gamma irradiated and non-irradiated nonwovens were significantly different, indicating that gamma rays did degrade the fibers. Moreover, the diameters of the fibers of non-irradiated and irradiated nonwovens were strongly affected after immersion in SBF, DMEM and artificial saliva for up to 30 days. Even if a considerable high dispersion in the fiber diameters exists before immersion in the different fluids, a significant decrease of the fiber diameter mean was observed for irradiated and non-irradiated nonwovens. The speed of diameter reduction was, nevertheless, dependent on the type of nonwoven. The decrease was obtained after 7 days on irradiated fibers, whereas it took 30 days of immersion to obtain similar diameter values on non-irradiated nonwoven (Figure 4). Among the tests carried out on non-irradiated nonwoven, the SBF solution triggered the most active degradation with a diameter reduction starting already after 7 days of immersion (Figure 4A).

Besides the results related to fiber’s diameters, our SEM examination provided useful qualitative morphological information about the appearance of the fibers in different treatment conditions. Here we showed an initial degradation process after 30 days of immersion in three different fluids. Non-irradiated nonwovens immersed in saliva showed an increased diameter from 7 to 15 days, possibly due to fiber hydration, which induced the swelling of fibers (Figure 2H) [40]. Degradation is a process influenced by fluid-induced relaxation of oriented polymer backbone chains. These structural changes allow the fiber´s coalescence, as observed in our work after 30 days of immersion in saliva (Figure 2I). However, similarly to what happened to fibers exposed to SBF and DMEM, diameters tended to enlarge at 15 days and were smaller at 30 days in saliva (Figure 2I). Regarding gamma irradiated nonwovens, diameters of membrane fibers immersed in SBF at 30 days (Figure 3C) were smaller relative to 7 days (Figure 3A) and 15 days (Figure 3B), suggesting that the thinner diameters at 30 days are compatible with an initial process of fiber’s degradation. An increase in fiber diameter from 7 to 15 days was observed after immersion in artificial saliva. Swelled fibers were also observed at 15 days after immersion in artificial saliva by Blasi et al. [40], which could be induced by fluid absorption into the fiber structure.

Another observed important event was the formation of cracks along the fibers immersed in SBF, DMEM and saliva after 15 days, probably due to the beginning of erosion induced by polymer degradation (Figure 3b,e,h). You et al. [38] reported that cracks were aroused along the fibers during the process of degradation. These erosions were more evidenced in the fibers of our membranes submitted to gamma radiation. This might be explained partially by the loss of molecular weight after gamma radiation. That decrease in the molecular weight after gamma radiation was previously related to polymeric chain scissions that are amplified with the fluids contact [22]. Additionally, Lee et al. [41] reported a decrease in the molecular weight of the tested polymers due to free radicals’ production and random chain scission after gamma irradiation. Indeed, it has been reported that gamma rays alter the physical properties of the bulk material [34] and can promote cross-linking and/or degradation (main chain scission) [34,42]. The cross-linking process increases the molecular weight due to the formation of new bonds in the main chains or the side groups [34,42]. Otherwise, the degradation process occurs when chain scission of a polymer results in a reduction in the molecular weight [34,42].

In our work, we monitored the fluid-induced degradation by the *M*_w_ of the PLGA in nonwovens immersed up to 30 days. No dramatic difference was observed between irradiated and non-irradiated nonwovens. Rediguieri et al. reported that the molecular weight of PLGA electrospun fibers decreased significantly by applying 20 kGy of gamma radiation or higher dosage, concluding that this effect seems to be dose-dependent [22]. They described that the *M*_w_ loss did not affect the morphology of the electrospun fibers up to 40 kGy, although a little narrowing of the fiber´s diameter was noted. In the present study, the dosage of 15 KGy was applied in accordance with the Center for Disease Control guideline for sterilization [43]. Accordingly, we observed that 15 kGy applied to our nonwovens did not affect the structural integrity of the fibers. Then, it seems an adequate dosage for future applications of our nonwoven structure in the field of oral mucosa tissue engineering.

As demonstrated by a decrease in *T_g_* observed by DSC analyses of non-irradiated and irradiated nonwovens in comparison with the respective controls, a plasticization (water absorption by the polymer chains) probably occurred in the bulk material depending on the immersion time. Li et al. [28] reported that a decrease in *T_g_* was related to an initial process of degradation caused by mass loss. Effectively, our GPC analyses showed a tendency of decreasing *M*_w_ in non-irradiated and irradiated nonwovens immersed in the different solutions in comparison with controls. This event is compatible with cleavage of the covalent bonds in the polymer backbone.

Artificial saliva was tested in order to reproduce a physiological environment for future applications in the field of regenerative medicine approaches for oral mucosa. It is noteworthy that few studies have used saliva for in vitro degradation tests [17,18]. Human saliva is composed of more than 99% of water, and electrolytes, such as sodium, potassium, calcium, magnesium, bicarbonate and phosphates [44]. In addition, it contains other components as immunoglobulin, proteins, enzymes, mucins and nitrogenous products, such as urea and ammonia [44], which contributes to oral tissues preservation and maintenance of the whole body health [45]. As discussed previously, morphological changes occurred on irradiated and non-irradiated electrospun fibers immersed up to 30 days in the tested fluids. All samples immersed in the fluids exhibited a decrease in fiber diameters (differences in means between immersed samples and controls are statistically significant for irradiated and non-irradiated samples for the three immersion fluids). For irradiated nonwovens, the maximum decrease of diameter was attained after 7 days of immersion whereas it took 30 days for non-irradiated samples in DMEM and saliva (Figure 4).

The electrospinning process generates highly oriented fibers. As shown in this work, PLGA used is a semicrystalline polymer, but the electrospun fibers have low crystallinity. Although the glass transition temperature found for the fibers was around 46 °C, which is not very close to the temperature the immersion fluids of the degradation/aging tests (37 °C), during the fiber–fluid contact, small molecules like water and fluid components can penetrate into the fibers causing an increase of chain mobility and, as a consequence, solvent/thermal induced crystallization processes take place. The crystallization processes can cause the fiber contraction, decreasing its diameter. Similar behavior was observed by Chu et al. [46] who stated that non-crystalline chains aggregate between the lamellar stacks produced by thermal induced crystallization and that the chains in the amorphous layers were highly constrained and prevented to undertake a relaxation process. As a consequence a small shrinkage percentage was observed.

In accordance with the SEM observations, degradation of non-irradiated nonwovens immersed in SBF for 7 days was also demonstrated by a DSC thermal event. In all the fluids, the enthalpy related to the endothermic event attributed to enthalpic relaxation increased after immersion (Table 2). The morphological changes were likely due to stress relaxation and chain break induced by the fluid.

In general, non-irradiated nonwovens immersed in the fluids presented a higher degree of crystallinity than irradiated ones, suggesting that a fluid-induced crystallization had higher influence over the higher molecular weight fibers. The higher degree of crystallinity may be associated with preferential degradation and solubilization of the amorphous phase, increasing the amount of the crystalline phase [40]. As observed for the non-irradiated nonwovens immersed in SBF for 30 days, irradiated nonwovens immersed in SBF for 30 days showed the endothermic event before *T*_g_ that can probably be due to a relaxation process of highly oriented chain domains induced by SBF. So far, no report on enthalpic relaxation process at temperature as higher than *T*_g_ (more than 20 °C in some cases) was reported yet for electrospun fibers, and other investigations must be carried out to clear the origin of this transition.

Electrospun polymers with high molecular weight like that used in the present study (*M*_w_ above 460,000) have long chains, being more susceptible to a drastic decrease when exposed to hydrolytic fluids [26]. In this regard, a strong decrease in the molecular weight of non-irradiated nonwoven immersed in saliva at 30 days (about 47%) was observed in comparison with the irradiated samples immersed in saliva at the same time point (about 1.8%), compared with the controls. Wu and Wang studied the effect of polymer MW on PLGA degradation [46]. PLGA 75/25 extruded rods (*M*_w_: 166,630; 241,450; 66,946; 31,403 and 10,876) were immersed in the buffer’s solutions for 72 days. Average *M*_w_ as a function of time during degradation for PLGA samples with the same composition and different molecular weight gives the constant of the degradation rate (k) of each sample. The absolute value of the degradation reaction rate increased with the increasing molecular weight of the polymers. In fact, the degradation process was hypothesized to occur in three phases: (i) first, a slow hydration of the polymers occurs within the first week; (ii) second, a fast degradation rate occurs within the second and the fifth weeks and (iii) third, the biodegradation rate slows down due to a lower molecular weight of the residual polymer, beginning near the sixth week [47].

We demonstrated that non-irradiated nonwovens immersed in the different solutions presented a higher degree of crystallinity (e.g., SBF 7 days, *X*_c_ = 23.7%) than the control (*X*_c_ = 3.7%). This occurrence suggests that a fluid induced crystallization took place during the ageing of the nonwovens. On the other hand, in the irradiated nonwovens, lower molecular weight allowed a faster kinetic of degradation [32]. DSC analysis of irradiated nonwovens showed higher *X*_c_ for nonwovens immersed for 30 days than the control, indicating that the amorphous phase of these nonwovens was degraded, becoming soluble, resulting in increasing the polymer crystalline phase. Saliva solution also induced an increase of OH stretching modes and additional bands variations in irradiated PLGA nonwovens with more similarity to SBF results when analyzed by FTIR spectroscopy. Additional bands were observed, which indicate polymer chemical changes and a likely higher degradation.

Finally, the PLGA (85:15) used in the present work usually degrades in six months [8]. This could explain why in our in vitro assay that lasted 30 days, it was difficult to conclude on the level of polymer degradation after immersion in SBF, DMEM and saliva solutions. However, SEM images of irradiated PLGA nonwoven immersed in saliva after 30 days indicated higher morphological changes (compared with non-irradiated) due to the coalescence of fibers. In addition, higher content of crystalline fraction detected by DSC and FTIR demonstrated additional bands attributed to acid end groups relative to polymer degradation.

## 5. Conclusions

This work reported that the electrospun PLGA scaffold in the form of a nonwoven presented an initial degradation process after gamma irradiation as demonstrated by SEM, DSC and GPC analyses. Notably, GPC analysis confirmed a strong decrease of molecular weight of irradiated nonwovens when compared to non-irradiated ones. Immersion in saliva for 30 days decrease about 40% of the PLGA molecular weight in the fibers of non-irradiated nonwoven. Our results demonstrated that electrospun PLGA 85:15 nonwovens were constituted by a semicrystalline material with moderate degradation properties after thirty days. The PLGA electrospun nonwovens present a preferential degradation of the amorphous fraction of the material increasing the crystalline content along the time. Based on mass loss after gamma radiation, the kinetics of degradation was slower on non-irradiated samples than on irradiated ones. Additionally, SBF showed a higher capacity of influencing crystallinity changes in both irradiated and non-irradiated PLGA nonwovens. Increased OH stretching modes and other additional bands indicated that SBF and saliva induced higher hydrolytic degradation of irradiated PLGA nonwovens. Overall, this work provides important results for future applications of PLGA nonwoven for oral mucosa tissue engineering.

## Figures and Tables

**Table 1:** Number (*M*_n_) and weight (*M*_w_) average molecular weight (*M*_w_) for non-irradiated PLGA nonwovens obtained in different in vitro degradation times.

Days	CONTROL		SBF		DMEM		SALIVA	
	*M*_n_	*M*_w_	*M*_n_	*M*_w_	*M*_n_	*M*_w_	*M*_n_	*M*_w_
0	253,900	460,400	-	-	-	-		
7	-	-	158,100 *	346,800	162,100 *	364,500	185,100	381,000
15	-	-	276,100	498,700	240,500	479,200	148,200	328,300 *
30	-		128,000 *	243,800	133,700 *	243,500	146,600	278,200 *

* Partial solubilization.

**Table 2:** Number (*M*_n_) and weight (*M*_w_) average molecular weights for irradiated PLGA nonwovens obtained in different in vitro degradation times.

Days	CONTROL		SBF		DMEM		SALIVA	
	*M*_n_	*M*_w_	*M*_n_	*M*_w_	*M*_n_	*M*_w_	*M*_n_	*M*_w_
0	75,000	156,300						
7			85,900	163,900	89,700 *	160,000	95,600	162,000 *
15			73,600	149,900	80,200	162,300	80,300	149,600 *
30			56,800	113,700 *	69,700	124,300 *	99,700	153,500 *

* Partial solubilization.

**Table 3:** Transition temperatures and enthalpies for non-irradiated PLGA nonwovens obtained in different in vitro degradation time.

Day	*T*_er_ (°C)/Δ*H_er_* (J/g)	*T*_g_ (°C)	*T*_cc_ (°C)	Δ*H*_cc_ (J/g)	*T*_m_ (°C)	Δ*H*_m_ (J/g)	*X*_c_ (%)
SBF							
7	68.5/12.2	44.8	96.4	9.56	151.2/182.4 ^a^	21.6/13.1	23.7
15	66.8/10.3	45.2	92.4	8.44	155.6	17.6	8.6
30	66/11.1	ND	91.4	15.6	55.1/174.5 ^a^	13.8/15.9	13.3
DMEM							
7	68.2/11.2	49.0	95.9	8.03	149.3/180.9 ^a^	12.6/16.2	19.6
15	68.7/12.9	40.8	94.7	15.6	152.1	14.4	4.1
30	67.9/18.4	ND	92.7	13.1	155.7	15.85	2.6
ARTIFICIAL SALIVA							
7	66.1/13.1	46.5	93.9	13.9	152.9	15.1	1.13
15	62.9/15.7	42.9	93.5	12.8	151.5	16.3	3.30
30	70.3/13.6	50.2	92.0	16.4	154.3	20.6	3.96

*T*_g_, glass transition temperature obtained from the second heating run; *T*_er_, *T*_m_ and *T*_cc_, enthalpic relaxation temperature, melting temperature and crystallization temperature on heating (cold crystallization); Δ*H*_er_, Δ*H*_m_ and Δ*H*_cc_, enthalpic relaxation, melting and cold crystallization enthalpies; ND, not determined and ^a^ bimodal peak. Non-irradiated PLGA control: *T*_er_ = 64.8 °C, Δ*H_er_* = 6.36 J/g, *T*_g_ = 45.9 °C, *T*_cc_ = 93.5 °C, Δ*H*_cc_ = 12.5 J/g, *T*_m_ = 152.0 °C, Δ*H*_m_ = 16.4 J/g and *X*_c_ = 3.7%.

**Table 4:** Transition temperatures and enthalpies for irradiated PLGA nonwovens obtained in different in vitro degradation times.

Days	*T*_er_ (°C)/Δ*H*_er_ (J/g)	*T*_g_ (°C)	*T*_cc_ (°C)	Δ*H_cc_* (J/g)	*T*_m_ (°C)	Δ*H*_m_ (J/g)	*X*_c_ (%)
SBF							
7	65.7/6.83	45.4	94.2	15.2	156.7	17.5	2.2
15	64.4/9.25	42.0	94.7	16.1	155.7	21.4	5.0
30	61.2/8.41	43.3	91.9	12.8	158.4	21.4	8.1
DMEM							
7	64.4/7.40	44.4	93.4	18.2	159.0	22.5	4.1
15	65.8/7.25	43.9	94.9	16.3	159.3	19.7	3.2
30	61.7/7.61	ND	89.9	10.6	157.9	17.8	6.8
ARTIFICIAL SALIVA							
7	63.8/7.79	43.2	92.1	15.6	157.8	20.2	4.3
15	63.9/16.3 ^a^	44.7	92.4	14.5	154.9	20.6	5.8
30	60.8/22.9 ^a^	ND	89.2	8.79	155.8	16.1	6.9

*T*_g_, glass transition temperature obtained from the second heating run; *T*_er_, *T*_m_ and *T*_cc_, enthalpic relaxation temperature, melting temperature and crystallization temperature on heating (cold crystallization); Δ*H*_er_, Δ*H*_m_ and Δ*H*_cc_, enthalpic relaxation, melting and cold crystallization enthalpies and ND, not determined and ^a^ bimodal peak. Irradiated PLGA control: *T*_er_ = 62.7 °C, Δ*H_er_* = 7.06 J/g, *T*_g_ = 48.8 °C, *T*_cc_ = 94.4 °C, Δ*H*_cc_ = 16.1 J/g, *T*_m_ = 156.6 °C, Δ*H*_m_ = 16.9 J/g and *X*_c_ = 0.8%.

## Supplementary Materials

The following are available online at https://www.mdpi.com/2073-4360/12/8/1853/s1.
