# Supplementary material for: In Vitro Degradation of Electrospun Poly(Lactic-Co-Glycolic Acid) (PLGA) for Oral Mucosa Regeneration
Source: Polymers (Basel). 2020 Aug 18;12(8):1853. doi: 10.3390/polym12081853 (PMC7465081; doi:10.3390/polym12081853)
Supplement: Supplementary file 1 [file polymers-12-01853-s001.zip › polymers-873520-supplementary.docx]

**Table S1. Composition of the SBF, DMEM and artificial saliva used in this work**

| Component | SBF  (mM) | DMEM  (mM) | Artificial Saliva (mM) |
| --- | --- | --- | --- |
| Na^+^ | 142.0 | 154.5 | 11.9 |
| K^+^ | 5 | 5.4 | 15.4 |
| Mg^2+^ | 1.5 | 0.8 | 0.53 |
| Ca^2+^ | 2.5 | 1.8 | 1.09 |
| Cl^-^ | 147.8 | 114.9 | 27.9 |
| HCO_3_^-^ | 4.2 | 44.0 | - |
| HPO_4_^2-^ | 1 | 0.9 | 2.09 |
| SO_4_^2-^ | 0.5 | 0.8 | - |
| D-glucose | - | 5.6 | - |
| L-Glutamine  Pyruvic-acid Na | -  - | 4.0  0.21 | -  - |
| Phenol-red.Na | - | 0.04 | - |
| Total Vitamins  Total Amino Acids | -  - | 0.03*  2.25* | -  - |
| Methylparaben | - | - | 6.8 |
| Liquid sorbitol | - | - | 136.7 |
| Carboximethyl cellulose | - | - | 31.6 |
| pH | 7.2-7.4 | 7.4 | 7.2 |

*Concentration of total amino acids and vitamins: g/L.
